# Direct comparison of size-dependent versus EpCAM-dependent CTC enrichment at the gene expression and DNA methylation level in head and neck squamous cell carcinoma

**DOI:** 10.1038/s41598-020-63055-y

**Published:** 2020-04-16

**Authors:** Martha Zavridou, Sophia Mastoraki, Areti Strati, George Koutsodontis, Apostolos Klinakis, Amanda Psyrri, Evi Lianidou

**Affiliations:** 1 Analysis of Circulating Tumor Cells Lab, Department of Chemistry, University of Athens, Athens, 15771 Greece; 2 Oncology Unit, 2nd Department of Internal Medicine - Propaedeutic, Attikon University Hospital, National and Kapodistrian University of Athens, Athens, Greece; 3 Biomedical Research Foundation Academy of Athens, Athens, 11527 Greece

**Keywords:** Biomarkers, Oral cancer

## Abstract

We directly compared two different approaches used for Circulating Tumor Cell (CTC) isolation, a size-dependent microfluidic system versus an EpCAM-dependent positive selection for downstream molecular characterization of CTC both at the gene expression and DNA methylation level in Head and Neck Squamous Cell Carcinoma (HNSCC). A size-dependent microfluidic device (Parsortix, ANGLE) and an EpCAM-dependent positive immune-magnetic isolation procedure were applied in parallel, using 10 mL PB from 50 HNSCC patients and 18 healthy donors. Total RNA was isolated from enriched CTCs and RT-qPCR was used to study the expression levels of *CK-19*, *PD-L1*, *EGFR*, *TWIST1*, *CDH2* and *B2M* (reference gene). Real time methylation specific PCR (MSP) was used to study the methylation status of RASSF1A and MLL3 genes. In identical blood draws, the label-free size-dependent CTC-isolation system was superior in terms of sensitivity when compared to the EpCAM-dependent CTC enrichment, since a significantly higher percentage of identical PB samples was found positive at the gene expression and DNA methylation level, while the specificity was not affected. Our results indicate that future studies focused on the evaluation of clinical utility of CTC molecular characterization in HNSCC should be based on size-dependent enrichment approaches.

## Introduction

Liquid biopsy provides a valuable source of biomarkers on prognosis and response to treatment of cancer patients^1^ and has recently shown a significant potential even for early cancer diagnosis and screening^2^. Isolation of circulating tumor cells (CTCs) from peripheral blood (PB) and their further downstream molecular characterization at the DNA, RNA and protein level is very important for reliable liquid biopsy analysis^3^. However, the identification and molecular characterization of CTCs is very challenging since these cells are extremely rare, and the amount of available sample for analysis in most cases is very limited^1,3^.

A variety of molecular assays have been developed for CTCs detection and molecular characterization. Molecular assays are based on total RNA isolation from CTCs and subsequent mRNA quantification of specific genes, and gDNA isolation for mutation analysis and DNA methylation studies^4^. CTC molecular characterization at the gene expression level has the potential to elucidate the critical signaling pathways involved in metastasis biology and even improve patient management. We have shown many years ago that the detection of *CK-19* expression in CTCs has prognostic significance in both early and metastatic breast cancer^5–7^. Beyond gene expression, DNA methylation analysis in CTCs has a high potential to provide novel epigenetic biomarkers for diagnosis, prognosis, risk assessment, and disease monitoring in many types of cancer^4^. Based on this, we selected two tumor-suppressor genes namely, *KMT2C/MLL3* and *RASSF1A* and evaluated their methylation status in CTCs of HNSCC patients. Recently, it was shown that down-regulation of lysine-specific methyltransferase 2 C (*KMT2C/MLL3*), a putative tumor suppressor leads to epigenetic and expression changes of DNA repair genes^8^. We also selected *RASSF1A*, since it has been shown that a high frequency of *RASSF1A* methylation is associated with more aggressive tumour phenotype among different cancer types^9^, while a recent meta-analysis suggested that there was a significant association between aberrant *RASSF1A* methylation and Head and Neck Squamous Cell carcinoma (HNSCC)^10^.

CTC-enrichment procedures are necessary prior to their molecular characterization, mainly for the reduction of background due to the presence of peripheral blood mononuclear cells (PBMCs). However, CTCs are highly heterogeneous and can have a different profile in different types of cancer. CTC heterogeneity and their phenotypic variation complicate their enrichment and subsequent phenotypic and molecular characterization^1,3^. Moreover, the effect of pre-analytical conditions and the establishment of quality control procedures in each analytical step is very important and very critical for CTC molecular characterization both at the gene expression and DNA methylation level^11^.

Over the past few years, a plethora of CTC isolation technologies based on their different biological and physical characteristics have been developed, including immune-magnetic, microfluidic, size dependent^12^ and function-based methods^13^. In the CellSearch^®^ system (Menarini Silicon Biosystems), the only Food and Drug Administration (FDA) approved approach for CTC detection and enumeration so far^14^, CTCs enrichment is based on a positive selection targeting EpCAM-positive cells. However, it is obvious that in all EpCAM-based CTC-capture systems, EpCAM-negative CTC subpopulations are non-detectable. This can have serious implications in cases where CTCs are characterized by a phenotypic plasticity that mainly reflects an epithelial to mesenchymal transition state (EMT)^15,16^. EMT results in down-regulation of epithelial markers like EpCAM and simultaneous up-regulation of mesenchymal markers^17,18^. The lack of expression of epithelial markers on CTCs due to EMT has resulted in the development of novel procedures for CTCs isolation that are based on label-free microfluidic devices, size-based filtration, or combination of microchips and positive selection through specific antibodies^1,3,13,19^. Antibody combinations on immunomagnetic beads when compared to anti-EpCAM antibodies enable capturing of a larger number of CTCs^20^.

Head and Neck Squamous Cell carcinoma (HNSCC) is a devastating disease and novel treatments are urgently needed. Molecular characterization of CTCs can be a powerful prognostic tool while serial assessments of CTCs at different time points during treatment may guide treatment decisions. EMT is a common phenomenon in HNSCC progression, thus EpCAM-based approaches can be suboptimal for CTC isolation. We have recently shown that in HNSCC the detection of CTCs overexpressing *PD-L1* provides important prognostic information^21^ and could be used for the selection of personalized medicine and treatment monitoring^22–24^.

The aim of the present study was to select the optimal enrichment system for CTC downstream molecular characterization in HNSCC. For this reason we directly compared the performance of a label-independent size-based microfluidic device versus an EpCAM-based CTC enrichment system using identical blood draws, and downstream molecular characterization both at the gene expression and DNA methylation level.

## Results

### RNA-based analysis

#### Comparison between size-dependent and EpCAM-dependent CTC-enrichment at the gene expression level

**Figure 1:**
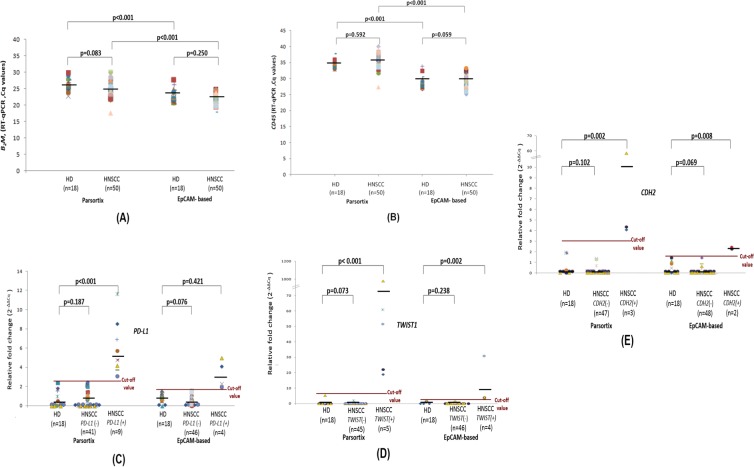
RT-qPCR results using size-dependent enrichment (Parsortix) and EpCAM-dependent CTC enrichment for: (**A**) Β2Μ (reference gene); (**B**) *CD-45* (specific leukocyte marker) (**C**) Relative fold change values (2^-ΔΔCq^) for reference gene (*Β2Μ*) and target gene (*PD-L1*) for healthy individuals and HNSCC patients’ CTC samples; (**D**) Relative fold change values (2^-ΔΔCq^) for reference gene (*Β2Μ*) and target gene (*TWIST1*); (**E**) Relative fold change values (2^-ΔΔCq^) for reference gene (*Β2Μ*) and target gene (*CDH2*) Red horizontal line: cutoff value. Different colors represent individual patients.

We first evaluated in a quantitative way the performance of these two different CTC enrichment approaches by downstream RNA-based CTC analysis run in parallel, using exactly the same procedure and the same RT-qPCR assays in material extracted from 50 HNSCC patients and 18 HD. Our results clearly reveal a significant difference in the expression levels of B2M (reference gene) in CTCs isolated through Parsortix versus EpCAM-dependent CTC enrichment. In all molecular assays, reference genes are selected on the basis that they are expressed at the same level both in the CTCs and in the PBMC fraction, as we have previously described 11,20. More specifically, as shown in Fig. 1A, Cq values for *B2M* were significantly lower in the EpCAM-based CTC-enrichment, indicating a higher number of *B2M* transcripts that definitely are due to a higher number of non-specific contaminant cells which are in their absolute majority PBMCs co-isolated with CTCs. B2M expression did not differ between HD and HNSCC within each CTC enrichment system as expected, since this reference gene is expressed at similar levels both in CTCs and PBMC, and the number of CTCs is very low (Fig. 1A). Our results also clearly reveal a significant difference in the expression levels of *CD45* (specific leukocyte marker) in CTC-fractions isolated through Parsortix versus EpCAM-dependent CTC enrichment. More specifically, as shown in Fig. 1B, Cq values for *CD45* were significantly lower in the EpCAM-based CTC-enrichment, indicating a higher number of *CD45* transcripts that definitely are due to a higher number of contaminant leucocytes co-isolated with the CTC fractions. CD45 expression did not differ between HD and HNSCC within each CTC enrichment system as expected (Fig. 1B).

#### Size-dependent CTC-enrichment

**Figure 2:**
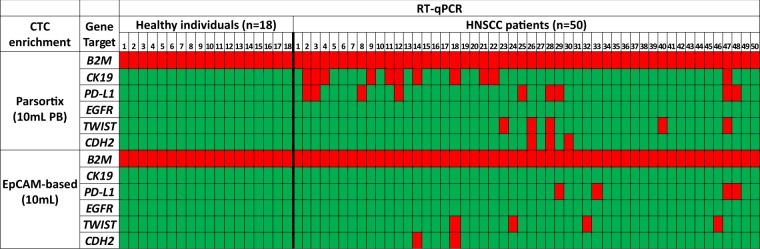
Direct comparison of gene expression in HNSCC and HD in CTC enriched using size-dependent enrichment (Parsortix) vs EpCAM-dependent CTC enrichment system using identical blood draws. (Red: gene expression, green: no expression).

All samples isolated with the size-dependent microfluidic device (Parsortix) were of excellent RNA quality as this was verified by our RT-qPCR assay for *B2M*. Relative fold change values (2^–ΔΔCt^) for *PD-L1* normalized according to *B2M* for individual samples are shown in Fig. 1B. Relative *PD-L1* expression in the Parsortix was 0.48 (range: 0–2.39) in HD, 0.71 (range: 0–2.48) in *PD-L1* negative HNSCC patients and 5.94 (range: 3.07–11.63) in *PD-L1* positive HNSCC patients. Relative fold change values (2^–ΔΔCt^) for *TWIST1*, and *CDH2* normalized according to *B2M* for individual samples are shown in Fig. 1C,D. Mean fold change of *TWIST1* expression in the Parsortix was 0.75 (range: 0–5.35) in HD, 0.10 (range: 0–2.04) in *TWIST1* negative HNSCC patients and 227.19 (range: 18.90–982.29) in *TWIST1* positive HNSCC patients. Mean fold change of *CDH2* expression in the Parsortix was 0.45 (range: 0–1.95) in HD, 0.13 (range: 0–1.35) in* CDH2* negative HNSCC patients and 22.26 (range: 4.08–58.49) in *CDH2* positive HNSCC patients. Our results for all genes tested are also shown in a heatmap (Fig. 2);* PD-L1* was overexpressed in 9/50 (18.0%) and *CK-19* in 11/50 (22.0%) cases. In 4/11 (36.4%) of *CK-19* positive samples *PD-L1* was also overexpressed. *EGFR* expression was not detected in any sample 0/50 (0.0%). Concerning EMT markers, *TWIST1* was overexpressed in 5/50 (10%), and *CDH2* in 3/50 (6%). None of 18 samples of the control group (HD) was found positive for mRNA expression for all genes tested.

#### EpCAM-dependent CTC enrichment

Using EpCAM-dependent CTC enrichment, all samples were also of excellent RNA quality as this was certified by RT-qPCR for *Β2Μ*. Relative fold change values (2^–ΔΔCt^) for *TWIST1* normalized according to *B2M* are shown in Fig. 1C. Mean fold change of *TWIST1* expression in the EpCAM-based enrichment was 0.92 (range: 0–3.16) in HD, 0.12 (range: 0–1.48) in *TWIST1* negative HNSCC patients and 10.63 (range: 3.2–31.12) in *TWIST1* positive HNSCC patients. Relative fold change values (2^–ΔΔCt^) for *CDH2* normalized according to *B2M* are shown in Fig. 1D. Mean fold change of *CDH2* expression in the EpCAM-dependent enrichment was 0.28 (range: 0–1.42) in HD, 0.07 (range: 0–1.41) in *CDH2* negative HNSCC patients and 2.29 (range: 2.25–2.33) in *CDH2* positive HNSCC patients. Mean fold change of *PD-L1* expression in the EpCAM-dependent enrichment was 0.84 (range: 0–1.54) in HD, 0.55 (range: 0–1.66) in *PD-L1* negative HNSCC patients and 3.24 (range: 1.72–4.99) in *PD-L1* positive HNSCC patients. Our results are shown for all genes tested in a heatmap (Fig. 2); *CK-19* and *EGFR* expression were not detected in any sample tested 0/50 (0%), while *TWIST1* was overexpressed in 4/50 (8%), and *CDH2* in 2/50 (4%) samples, *PD-L1* overexpression was detected in 4/50 (8%) cases. None of 18 samples of the control group (HD) was found positive for mRNA expression for all genes tested.

In HNSCC *CK-19* positive CTCs were only detected when enriched using the label-independent Parsortix approach but not in the EpCAM-dependent immunomagnetic CTC enrichment (Fig. 2). *PD-L1* overexpression was detected in 9/50 (18.0%) cases after using the Parsortix device but only in 4/50 (8.0%) cases after using EpCAM-dependent CTC-enrichment. Three (#29, #47 and #48) out of four samples found positive for *PD-L1* overexpression after using EpCAM-based CTC enrichment were also positive after size-dependent enrichment. CTCs are heterogeneous, so it is possible that in these patients there were populations of *PD-L1* positive CTCs that were both EpCAM+ and EpCAM-. *EGFR* mRNA was not detected in any sample tested, using both approaches.

### DNA methylation analysis

#### Size-dependent CTC-enrichment

Ten peripheral blood samples from healthy donors were processed following exactly the same procedure as the HNSCC samples in order to verify the diagnostic specificity of the DNA methylation analysis assays. None of the HD samples was found positive for *RASSF1A* and *MLL3* methylation (0/10, 0%). DNA methylation analysis was performed in 43 samples isolated using the Parsortix microfluidic device; RASSF1A methylation in 3/43 (7%) and MLL3 methylation in 7/43 (16.3%) samples.

#### EpCAM-dependent CTC enrichment

Ten peripheral blood samples from healthy donors were processed following exactly the same way as the HNSCC samples in order to verify the diagnostic specificity of the DNA methylation analysis assays. None of the HD samples was found to be positive for *RASSF1A* and *MLL3* methylation (0/10, 0%). DNA methylation analysis was performed in 31 samples isolated using the EpCAM-dependent immunomagnetic enrichment; *RASSF1A* in 0/31 (0%) and *MLL3* promoter methylation in 4/31 (12.9%).

#### Direct comparison between size-dependent and EpCAM-dependent CTC-enrichment for DNA methylation analyses

**Figure 3:**
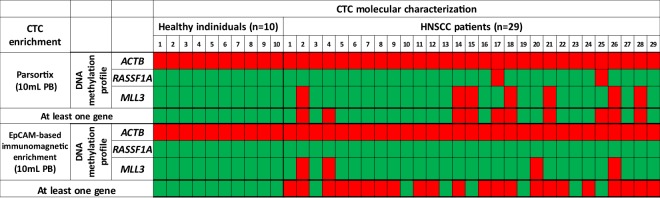
Direct comparison of DNA methylation analysis markers in HNSCC (n = 29) and HD (n = 10) in CTC enriched using a size-dependent enrichment (Parsortix) vs EpCAM-dependent CTC enrichment system, using identical blood draws. (Red: positive for DNA promoter methylation, green: negative for DNA promoter methylation).

For 29 of these patients we had available material for a direct comparison between size-based and EpCAM-dependent CTC-enrichment at the DNA methylation level. The assessment of correlation for DNA methylation profile of each gene of interest between CTCs isolated using the label-independent versus the EpCAM-dependent CTC-enrichment method was performed using the χ^2^ test for these 29 matched samples. We did not observe any statistically significant correlation for the above samples in none of the tumor-suppressor genes tested (Fig. 3).

## Discussion

CTC enrichment technologies based on EpCAM present the risk of missing EpCAM-negative CTCs. Recent technological advances have now enabled CTC-enrichment based on different biological and physical properties of CTCs. Label-independent enrichment microfluidic devices for CTC enrichment have the potential to isolate successfully CTCs in an EMT state, since EMT leads to down-regulation of EpCAM.

In order to evaluate the most appropriate system for downstream molecular characterization at the gene expression and DNA methylation analysis level in CTCs from HNSCC patients, we performed for the first time a direct comparison study, using identical blood draws, between a label-independent size-based microfluidic device and an EpCAM-based CTC enrichment system. More specifically, we studied the expression levels of *CK-19*, *PD-L1*, *EGFR*, *TWIST1*, *CDH2* and *B2M* (reference gene) and the methylation status of *RASSF1A* and *MLL3* genes, that are all indicating the presence of CTCs. Our data clearly indicate that the CTCs population enriched using the label-independent size-based microfluidic device is of higher purity than that of CTCs isolated using EpCAM-based CTC enrichment; *Β2Μ* levels revealed a much lower PBMC contamination in the size-dependent approach compared to EpCAM-dependent CTC enrichment. Our results were in concordance with two recent studies; Obermayer *et al*. reported that the Parsortix system was more appropriate to remove leukocytes and allow for the subsequent molecular analysis in a high purity of the enriched cells^25^. The Parsortix system was also evaluated as having the highest recovery rate and the lowest leukocyte contamination, compared to two different CTC isolation methods^26^.

Our results indicate that distinct populations of CTCs are isolated when these two different enrichment approaches are used. It is clear though that when the size dependent isolation was used a lot more positive events for the presence of CTCs are detected, eg the same sample is characterized as CTC negative using EpCAM isolation. We report a higher percentage of *PD-L1* positive samples following CTC isolation with the Parsortix system in comparison to the EpCAM-dependent approach. Three out of four samples found positive for *PD-L1* overexpression after using EpCAM-dependent CTC enrichment were also positive after using the Parsortix device. The clinical significance of this finding will be soon evaluated in an independent study, involving a large number of HNSCC patients under specific treatments. According to a recent meta-analysis, a high *PD-L1* expression in the tumour cells did not correlate with poor prognosis of patients suffering for oral squamous cells carcinoma^27^. Most studies published on *PD-L1* expression are performed in the primary tumour and are based on immunohistochemistry, and have shown a significant variation in results, limiting the use of PD-L1 expression by immunohistochemistry as a prognostic biomarker in clinical practice^27^. Our results on *PD-L1* mRNA overexpression in CTCs after Parsortix enrichment indicate towards a clinical evaluation of this finding in a large number of clinical samples.

*EGFR* mRNA was not detected in any sample tested, using both approaches. We did not observe any correlation in the expression of EMT markers (*TWIST1* and *CDH2)* between the Parsortix and EpCAM-dependent CTC enrichment. According to our results, the number of patients positive for *CDH2* expression is very low, therefore, to draw any conclusion studying this gene is inappropriate, so we simply report our finding.

In HNSCC several preliminary studies have detected CTCs by using various enrichment-isolation methods that yielded varying results with respect to the number of CTCs and the frequency of patients with positive CTCs^28,29^. In our recent study^21^ when we evaluated the expression of *PD-L1* in CTCs of HNSSC patients using EpCAM isolation, we did not find any *CK-19* mRNA expression in CTCs (data not shown). Similar to our results, Bozec *et al*. have shown that according to the results obtained by the CellSearch system, CTCs are in a relatively low proportion in patients with locally advanced HNSCC^29^. Thus, HNSCC is not considered as an exclusively EpCAM positive type of cancer.

In CTC samples isolated using the Parsortix microfluidic device a significantly higher percentage of samples positive for *MLL3* and *RASSF1A* promoter methylation was detected when compared to paired samples isolated using the EpCAM-positive immunomagnetic approach. *RASSF1A* and *MLL3* were found to be methylated in CTCs at various percentages, confirming the presence of heterogeneity, even in CTCs isolated from the same patient.

There is an urgent need for the evaluation of technologies investigating the expression, mutation and DNA methylation status of CTCs and it is important to select the best system in every type of cancer. Lampignano *et al*. developed a workflow using the Parsortix system for single CTC analysis, permitting for the first time assessment of the heterogeneity of *PIK3CA* mutational status within patient-matched EpCAM^*high*^ and EpCAM^*l*^*°*^*w/negative*^ CTCs^30^. Furthermore, Gorges *et al*. established reliable workflows in order to study multi-marker profiles of single CTCs by qPCR approaches. These workflows were combined with Parsortix system enabling the recovery of higher quality RNA^31^. El-Heliebi *et al*. compared three different CTC isolation systems for gene expression and DNA mutation analysis in CTCs of prostate cancer patients and showed also differences^32^. The incorporation of microfluidics into CTC isolation is now emerging for clinical applications^33^. Many microfluidic technologies have reported high sensitivity and specificity for capturing CTCs, however, the question still remains as to the superiority in comparison to immunoaffinity based approaches, specifically to identify different CTC populations^34^.

In conclusion, distinct populations of CTCs are isolated with these two different enrichment technologies. It is clear though that when the size dependent isolation was used a lot more positive events for the presence of CTCs are detected. The clinical significance of this finding will be soon evaluated in an independent prospective study, involving a large number of HNSCC patients under specific treatments.

## Materials and Methods

**Figure 4:**
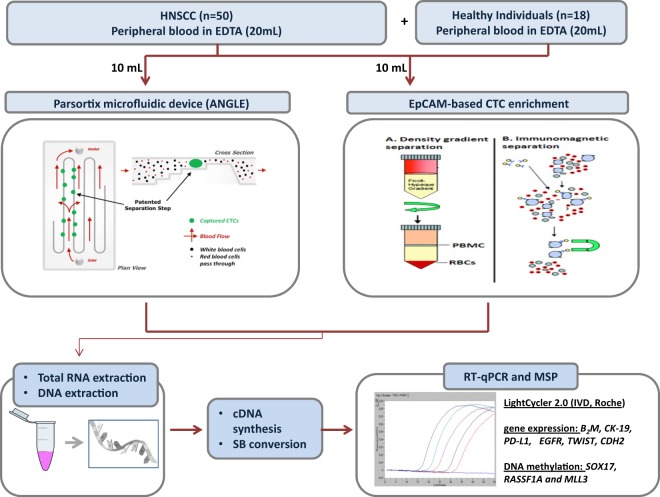
Experimental flowchart of the study.

The experimental flowchart of the study is outlined in Fig. 4.

### Peripheral blood samples collection

Fifty patients with squamous cell carcinoma of the oral cavity, oropharynx, hypopharynx or larynx and eighteen healthy donors were included in this study. Two of these patients, heavy smokers, were positive for HPV. In oral cavity and hypopharynx no HPV testing is indicated, while in some oropharynx cases HPV testing is not feasible because we have only cytology. Blood specimens were obtained before initiation of treatment. The first 5 ml of blood were discarded to avoid skin epithelial cells contamination. Peripheral blood was then collected into two 10 mL K_2_EDTA tubes (BD Vacutainer, Plymouth, UK) and mixed immediately after blood draw by inverting gently 10 times, maintained at room temperature (RT) and processed within 3 h, to CTC isolation. A size-dependent microfluidic device (Parsortix, ANGLE plc, UK) and an EpCAM-dependent positive immunomagnetic enrichment procedure were applied simultaneously, using 10 mL PB in each case. All patients gave their informed consent for participating in this study. IRB approval for the collection of samples was obtained from Attikon University Hospital, National and Kapodistrian University of Athens, Greece, (EΒΔ472/30–10–14). Attikon University Hospital approved the research and all methods were performed in accordance with the relevant guidelines and regulations.

### CTCs enrichment using a size-based microfluidic device

The Parsortix CE-marked system (ANGLE plc, UK), based on micro-fluidics^35^ was used to capture and then harvest CTCs from 10 mL whole blood collected in K_2_EDTA tubes. Separation of blood components took place in a microscope slide sized disposable cassette, which contains a series of steps leaving a 6.5 μm measuring gap between the top cover and the final step^36–38^. Following enrichment, CTCs were harvested in a total volume of 200 μL of PBS deposited into 1.5 mL Eppendorf tubes by applying a reverse flow to the cassette using a specific software protocol. Spiking experiments were performed using 100 cells spiked in 10 mL peripheral blood of a healthy donor using the SCC-47 NHSCC cell line. According to our results, b2M (reference gene) and *CK-19* (epithelial marker) transcripts were quantified in these cells before and after spiking. *B2M* and *CK-19* trancripts were detected in these spiked samples after enrichment using the Parsortix.

### CTCs enrichment using EpCAM-dependent immunomagnetic approach

Magnetic beads, coated with the monoclonal antibody BerEP4 against the human epithelial antigen, EpCAM, were used for CTCs enrichment (Dynabeads® Epithelial Enrich, Life Technologies, USA) from 10 mL whole blood collected in K_2_EDTA tubes as previously described^21,39^.

### RNA extraction and cDNA synthesis

Total RNA from CTCs was isolated using TRIZOL-LS (ThermoFisher Scientific, USA), followed by cDNA synthesis as previously described^21^.

### RT-qPCR

We used our previously developed and analytically validated RT-qPCR assays for *PD-L1*, keratin 19 (*CK-*19), epidermal growth factor receptor (*EGFR*) and beta-2-microglobulin (*Β2Μ*) (used as a reference gene)^7,40^. A multiplex RT-qPCR was performed for the quantitative determination of EMT markers, cadherin 2 (*CDH2*), and TWIST family transcription factor 1 (*TWIST1*) as previously reported^41^. All RT-qPCR reactions were performed in the LightCycler^®^ 2.0 (IVD instrument, Roche Diagnostics, Germany) following the MIQE guidelines^42^. The amplification reaction mix for *B2M* contained 1 μL of the PCR Synthesis Buffer (5Χ), 1.2 μL of MgCl_2_ (25 mM), 0.15 μL dNTPs (10 mM), 0.3 μL BSA (10 μg/μL), 0.1 μL Hot Start DNA polymerase (HotStart, 5 U/μL, Promega, USA), 0.25 μL of forward and reverse primer (10μΜ), 0.83 μL of hydrolysis probe (3 μM). 1 μL of cDNA was added in the PCR mix and dH_2_O was added to a final volume of 9 μL. Protocol conditions: 1 cycle at 95 °C for 2 min, followed by 45 cycles of: 95 °C for 10 s, annealing at 58 °C for 20 s and extension at 72 °C for 20 s, and a final cooling cycle at 40 °C for 30 s. In each RT-qPCR run we used the same cDNA as a positive control in order to evaluate the accuracy and reproducibility of the results. For this purpose we aliquoted cDNA from MCF-7 cells and then stored these aliquots at −80 °C. The expression levels of *PD-L1*, *CDH2*, and *TWIST1* were normalized using the 2^−ΔΔCt^ approach in respect to the expression of *Β2Μ*^43^.

### gDNA isolation from CTCs

gDNA was extracted from CTCs using the TRIZOL-LS reagent (ThermoFisher Scientific, USA) as previously described. Isolated gDNA^44^ was dissolved in 30 μL of 8 mmol/L NaOH.

### Sodium Bisulfite (SB) treatment

gDNA samples were treated with SB, to convert all non-methylated cytosines to uracil, while methylated cytosines were not converted, using the EZ DNA Methylation Gold Kit (ZYMO Research, USA). SB-treated DNA was stored at −70 °C until use. In each SB reaction, dH_2_O and 100% methylated DNA were included as negative and positive control respectively.

### Real-time MSP

We used our previously designed and analytically validated real time MSP assays for each gene of interest. All experiments for *RASSF1A*^45^ methylation analyses were performed in the LightCycler 2.0 (*IVD* instrument, Roche, Germany), whereas *MLL3* methylation analysis was performed in the 96-well plates of LightCycler^®^ 480 system (IVD, Roche Molecular Diagnostics, Switzerland) in a total volume of 10 μL. We report a sample as methylation positive, when we detect an MSP amplification signal (Cq<40.00) and as methylation negative only in the complete absence of amplification signal.

### Quality control

Quality control checks were performed in all steps prior to sample analysis. In each step of the analytical procedure we included appropriate positive and negative controls in order to ensure the quality and reproducibility of results as previously described^11^. Before proceeding to the SB-treatment and real-time MSP, we assessed the gDNA integrity of all samples by amplifying the *PIK3CA* exon 20. Only samples that were positive for *PIK3CA* exon 20 amplification were further processed to SB treatment. After SB-treatment, converted DNA was also checked by a real-time PCR assay for β-actin (ACTB) and samples that were not amplified were excluded from the study^11,46^. Human placental genomic DNA (gDNA; Sigma-Aldrich, USA) was used as a real-time MSP negative control after SB-treatment, while Universal Methylated Human DNA Standard (ZYMO Research, USA) was used as fully methylated (100%) positive control.

### Statistical analysis

We performed statistical evaluation of data using SPSS (SPSS Statistics 25.0). We used the chi-square test of independence, and the Mann Whitney test (SPSS, version 25.0) to make comparisons between groups. A level of P < 0.05 is considered statistically significant.

## Conclusions

Our data indicate that molecular characterization of CTCs based on a label-free size-dependent isolation CTC system, gives superior results compared to the EpCAM-dependent approach, in HNSCC patients. The clinical significance of CTC detection using this approach remains to be elucidated in prospectively collected well-defined patient cohorts. Our results indicate that future studies focused on the evaluation of clinical utility of CTC molecular characterization in HNSCC should be based on size-dependent enrichment approaches.
